# Tolerance to occasional frosts during germination in Chilean Altiplano quinoa (*Chenopodium quinoa* Willd.) cultivars

**DOI:** 10.3389/fpls.2025.1718308

**Published:** 2026-01-26

**Authors:** Ignacio Delfino Yurin, José Delatorre-Herrera, Juan Pablo Rodríguez, José Pablo Delatorre-Castillo, Isabel Sepúlveda-Soto, Cristopher Low-Pfeng

**Affiliations:** 1 Fundación para la Innovación Agraria, Santiago, Chile; 2 Universidad Arturo Prat, Faculty of Renewable Natural Resources, Agricultural Research Nucleus for Extreme Environments, Iquique, Chile; 3 University of Rostock, Faculty of Agriculture, Civil and Environmental Engineering, Rostock, Germany

**Keywords:** quinoa, occasional frost, seed germination, germination phases, osmotic adjustment

## Abstract

Quinoa (*Chenopodium quinoa* Willd.) is increasingly cultivated in marginal environments; however, early-season frosts pose a major constraint to successful stand establishment. Despite evidence of genotypic variation in cold tolerance, the effects of short, occasional frost events occurring at specific germination stages remain poorly understood. We evaluated the impact of single, occasional frost events (0, −2, and −4 °C) applied during distinct germination phases: Phase I (imbibition, 4 h), Phase II (absorption, 2 h), and Phase III (radicle protrusion, 6 h), in two Chilean quinoa cultivars (Roja and Amarilla). Across four replicates (50 seeds per cultivar per treatment), we quantified germination percentage and rate, imbibition rate, osmotic potential (Φ), and proline content under controlled conditions. The main results were: Frost exposure during Phases I and II markedly reduced final germination, frequently causing reductions greater than 70% relative to the control, with the strongest inhibition observed at −4 °C during Phase I. In contrast, when frost was applied during Phase III, germination was less affected and generally remained above 50%, indicating clear phase-dependent tolerance. Frost conditions reduced imbibition rate and induced a more negative osmotic potential, accompanied by increased proline accumulation, particularly at −4 °C during Phases II and III. Amarilla showed higher germination than Roja when frost was applied during Phase III, revealing cultivar-specific responses. These findings demonstrate that the first hours following sowing constitute a critical sensitivity window to occasional frost in quinoa. Phase-specific physiological responses, including osmotic adjustment, appear to play a protective role under freezing stress. The observed cultivar differences highlight exploitable genetic variation and provide valuable information for improving sowing management and breeding strategies under increasing climatic variability.

## 1 Introduction

Quinoa (*Chenopodium quinoa* Willd.) is an Andean grain traditionally cultivated in the Andean highlands of Peru, Bolivia, Ecuador, and Chile, is increasingly being produced worldwide due to its nutritional value and its ability to adapt to marginal environments, for this reason more countries try to cultivate this crop (Bazile et al., 2016; Jaikishun et al., 2019; García-Parra et al., 2020; Alandia et al., 2020; Rodríguez et al., 2020; Delgado et al., 2025). Like *Chenopodium pallidicaule* (Rodriguez et al., 2016), the species is resilient to abiotic stresses, such as drought, salinity, and low temperatures. For instance, the Real quinoa variety can tolerate frosts down to −5.3°C at the sixth leaf stage and other cultivars can resist to -8°C trough structural modifications like generation of secondary branches (Jacobsen et al., 2005; Achallma Ccoriñahui, 2025). Recently, quinoa has been recognized as a strategic crop in the context of climate change thanks to advances in our understanding of its physiological and molecular mechanisms of stress tolerance (Rosa et al., 2009; Choukr-Allah et al., 2016).

Frost events are common in the Andean altiplano of Bolivia and Chile, where there can be more than 200 frost days per year (Moreno et al., 2009; Winkel et al., 2009; Herzog et al., 2011; Vommaro and Bidaseca, 2021). There are two main types of frost are recognized: radiative (“white frost”), which is characterized by high humidity and the deposition of ice on plant surfaces; and advective (“black frost”), which is caused by cold, dry air masses that freeze surface water without condensation (Squeo et al., 1991; Sierra-Almeida et al., 2009). Depending on timing, duration, and intensity, both types can severely damage crops regardless of the stage of growth (Snowball et al., 2022). Climate variability has increased the unpredictability of such events, creating additional risks for crop establishment (IPCC, 2021).

Seed germination is a critical stage in the establishment of plants and comprise three phases: rapid water uptake (Phase I), metabolic activation (Phase II), and radicle protrusion (Phase III) (Jojoa et al., 2021; Ghadirnezhad Shiade et al., 2024). Cold stress during imbibition can impair membrane integrity, reduce water absorption, and slow metabolism (Bedi and Basra, 1993; Bois et al., 2006). Short frost events at −1°C for a few hours can freeze intracellular water, compressing the starch granules affecting the crystalline structure, reduce enzymatic activity, and cause embryo necrosis upon thawing (Garg et al., 2025). Experimental evidence shows that germination responses vary widely among species. For example, in maize (*Zea mays* L.), chilling at 0°C for three hours primarily damages the radicle during Phase III. In contrast, in subterranean clover (*Trifolium subterraneum* L.), a similar exposure affects all germination phases due to blocked water entry through the micropyle (Mendizábal et al., 2011), all this response are relationated with four areas: phenotype, physiology, hormone response and signaling pathways (Yu et al., 2025).

Quinoa seeds generally germinate efficiently between 6 and 17°C, reaching nearly 100% germination within the first 8 hours (Ceccato et al., 2015; Delatorre-Herrera and Pinto, 2009). However, at suboptimal temperatures (0–5°C), germination is reduced by ~60% (Jacobsen and Bach, 1998). At sub-zero temperatures, extracellular ice formation alters osmotic gradients, increasing respiratory demand and enzymatic activity (Wang et al., 2025). This is followed by a transient metabolic arrest (Nijsse et al., 2004). Quinoa can tolerate a wide thermal range of −8°C to 35°C, but its germination performance depends on genotype, developmental stage, and environmental humidity (Jacobsen et al., 2007; Ramírez-Santiago et al., 2020). Recent research confirms that highland ecotypes exhibit greater tolerance to nocturnal frosts (down to −8°C), whereas inter-Andean valley accessions are more sensitive (to −4°C) (Rosa et al., 2009; García-Parra et al., 2020).

At the molecular level, cold stress during imbibition has been associated with altered expression of aquaporins, ROS-scavenging enzymes, and hormonal regulators, such as abscisic acid (ABA) and gibberellic acid (GA) (Hao et al., 2022). In soybeans, cold-water imbibition has been linked to increased membrane leakage and delayed radicle emergence. It has also been associated with the identification of genomic loci for cold tolerance (Haidar et al., 2023). Furthermore, Suo et al. (2024) demonstrated that the combined effects of low temperature and high soil moisture significantly reduce germination success in sensitive crops, highlighting the complexity of environmental interactions. Most of the available background on tolerance to occasional frost in quinoa comes from studies conducted on different developmental stages of the plant rather than on seeds or pre-germinative phases. Delfino Yurin (2008) showed that the Roja selection exhibits higher survival and reduced structural damage compared with the Amarilla selection when exposed to −6°C. Although these results do not directly assess germination processes, they clearly reveal genotypic differences in freezing tolerance that may also manifest during the earliest stages of the life cycle. Both cultivars used in this study originate from highland quinoa populations (~3800 m a.s.l.) but have been propagated for several generations under desert lowland conditions (~1000 m a.s.l.), where different pathways of local adaptation may have shaped divergent physiological responses to freezing stress.

In this context, we hypothesized that, despite sharing a highland origin, the Roja cultivar has retained or developed greater tolerance to occasional frost events during germination than the Amarilla cultivar. This study evaluated the effects of frost intensity and duration (between −4°C and 0°C) on water uptake and the different germination phases in both cultivars, contributing to a better understanding of quinoa’s resilience under cold stress.

## 2 Materials and methods

### 2.1 Experimental site and plant material

The study was conducted at the Laboratory of Agriculture for Extreme Environments, Arturo Prat University, Huayquique Campus (20°11′ S; 70°09′ W), in the Tarapacá Region of Chile. This area is characterized by a coastal desert climate, with average annual temperatures of around 18°C and no occurrence of natural frost. It is located at an altitude of 20 meters above sea level.

These seeds are from a cultivar of two ecotypes that were brought from the Chilean Altiplano at 3800 meters above sea level and multiplied at the Canchones Experimental Station (1000 meters above sea level). In their area of origin (altiplano), they are sown from September to October when the average minimum temperature is between 1.9 and 5.7 °C. However, there are occasional frosts with temperatures below 0 °C throughout the growing season (September to January) (Salinas et al., 2008). Prior to germination, the seeds were equilibrated of ~12% moisture content by oven-drying at 30 °C, to standardize initial seed water content.

The control group seeds were tested at 20 °C and showed more than 90% germination in less than four hours. Therefore, it was deemed unnecessary to perform viability tests with 2,3,5-triphenyl tetrazolium chloride (TTC). After each low-temperature exposure, the seeds were returned to 20 °C (the optimal conditions) and monitored until 100% germination was reached by the control group. Thus, viability was defined as the ability to germinate under optimal conditions after frost treatment, consistent with international seed-testing standards that consider standard germination as the primary viability test for non-dormant seed lots (ISTA, 2024; AOSA/SCST, 2010; Moore, 1985).

### 2.2 Equipment and experimental conditions

Germination and frost treatments were performed using precision incubators (Precision Scientific model 815; Binder WTC model D-78502). The relative humidity inside the chambers ranged from 53 and 60%. Seed weights were recorded using analytical balances (Sartorius, models BLI 5005 and MC1).

### 2.3 Determination of imbibition curve and germination phases

A preliminary experiment was conducted to determine the imbibition curve, water content dynamics, and duration of the germination phases for both quinoa cultivars. One hundred seeds per cultivar were placed in four Petri dishes (n=4), each containing 10 mL of distilled water. Seed weight and germination counts were recorded every hour until 100% germination was achieved. The duration of the three germination phases was estimated as follows: Phase I (imbibition): 4 hours, Phase II (absorption): 2 hours; and Phase III (radicle protrusion): ≥ 6 hours.

### 2.4 Determination of lethal temperature and exposure time for each germination phase

Quinoa seeds were placed in Petri dishes containing a 1:1:1 mixture of peat, sand, and perlite. Then, 25 grams of the mixture were added to each dish, and the dishes were then moistened with 20 mL of distilled water. This process was carried out using a 20 ml volumetric pipette. To prevent humidity between the Petri dishes and the cold chamber atmosphere, the dishes were covered. The relative humidity within the chamber ranged from 45% to 49%, while the humidity within the dishes ranged from 53% to 58%. These conditions simulated a “black frost,” in which the substrate temperature remains above the air dew point, avoiding condensation and allowing ice to form exclusively on the substrate surface.

The effect of low temperatures was evaluated for each of the three germination phases (4, 2, and 6 hours, respectively) under optimal conditions (20 °C). For each phase, 50 seeds of the Roja and Amarilla cultivars were exposed to frost at 0, −2, and −4 °C for the corresponding duration. Then, they recovered at 20 °C until 100% germination was reached under control conditions.

Specifically, during Phase I (imbibition), the seeds were exposed directly to temperatures of 0 °C, −2 °C, and −4 °C for four hours and then transferred to 20 °C. In Phase II (the absorption phase), the seeds were first germinated at 20 °C for four hours (the Phase I duration). Then, they were exposed to 0, −2, or −4 °C for two hours. Afterwards, they returned to 20 °C. In Phase III (radicle protrusion), the seeds underwent pre-germination for six hours, 20 °C (Phases I and II). Then, the seeds were exposed to 0 °C, −2 °C, or −4 °C for six hours. Subsequently, the seeds were returned to an optimal temperature for the remainder of the experiment.

Seeds failed to germinate at −5 °C (0% for the Amarilla cultivar, 4% for the Roja cultivar). Based on these results, the lethal temperature range for subsequent experiments was set at −4 °C, −2 °C, and 0 °C (See **Supplementary Material**).

### 2.5 Frost treatments during different germination phases

The seeds were germinated in Petri dishes containing a substrate mixture of peat, sand, and perlite (ratio 1:1:1; 25 g per Petri dish), every Petri dish was moistened with 20 mL of distilled water. The plates were sealed with parafilm to maintain substrate humidity (53%–58%) independently of chamber humidity (45%–49%), which simulated a “black frost” condition (freezing of surface water without condensation).

Viability after each frost exposure was assessed following the procedure described above, with seeds returned to 20 °C to complete germination under optimal conditions.

According to the previously determined imbibition curve, frost treatments were applied to each germination phase: Phase I (4 hours), Phase II (2 hours), and Phase III (6 hours) (**Table 1**). For each phase, 50 seeds per cultivar (four replicates) were exposed to −4 °C, −2 °C, or 0 °C during the corresponding phase. After the frost treatment, seeds from Phases I and II were returned to 20 °C to complete germination, whereas in Phase III germination progress and radicle viability were assessed directly after frost exposure.

**Table 1:** Duration of frost treatments (0 °C, −2 °C, and −4 °C) applied to each.

Germination phase tested	Temperature applied (°C)	Exposure duration	Cultivar	Experimental condition
Phase I – Imbibition	0, −2, −4	4 h	Roja/Amarilla	Conducted independently (Phase I only exposed to frost)
Phase II – absorption	0, −2, −4	2 h	Roja/Amarilla	Conducted independently (Phase II only exposed to frost)
Phase III – Radicle protrusion	0, −2, −4	6 h	Roja/Amarilla	Conducted independently (Phase III only exposed to frost)

### 2.6 Measured variables

The following variables were evaluated in quinoa seeds from both cultivars during each germination phase: germination percentage, germination rate, and imbibition rate. The germination rate (GR) was calculated using Equation 1:

(1) Germination rate= ∑​ni/t 

where n_i_ is the number of germinated seeds in the treatment and t is the time elapsed from sowing until the last seed germinates. The imbibition rate was quantified as the difference in seed weight between the initial and final stages of each germination phase.

The proline content of the seeds from both cultivars was determined after exposure to low temperatures using the method of Bates et al. (1973). In brief, 0.5 g of seeds were freeze-dried for 24 hours and ground into a fine powder with a mortar and pestle. Then, a 100 mg sample was extracted in 1 mL of 3% sulfosalicylic acid (v/v) and centrifuged at 10,000 rpm for 10 minutes at 4 °C. The resulting supernatant was treated with acetic acid and acidic ninhydrin, then incubated at 90 °C for one hour and cooled on ice. The colored phase was extracted with toluene, and the absorbance was measured at 520 nm using a UV-Vis spectrophotometer (Biomate-5). Quantification was performed using a calibration curve prepared with six dilutions of proline standards ranging from 20 to 120 μg mL^-1^.

The osmotic potential (Φ) was measured at the end of each germination phase for each temperature treatment. To do so, 0.1 g of freeze-dried seeds were macerated at −75 °C in a mortar in 1 mL of nanopure water. The mixture was then centrifuged, and a 200 µL aliquot of the supernatant was analyzed in an Advance model 3000 osmometer. The osmotic potential was calculated using the Van’t Hoff equation (Equation 2):

(2) Φ=C∗T∗R 

where C is the concentration or osmolality (mOsmol/kg), T is the absolute temperature, and R is the gas constant (0.00831 MPa mol^-1^ K^-1^).

### 2.7 Statistical analysis

All statistical analyses were conducted using the complete dataset under a factorial structure. The fixed factors were cultivar (Roja, Amarilla) and temperature (0 °C, −2 °C, −4 °C), including their interaction term. Each germination phase was analyzed independently using this design.

An analysis of variance (ANOVA) was applied to germination assays, and a covariance analysis (ANCOVA) to the vegetative-stage variables, considering the measurement days as covariates. All analyses were performed using InfoStat v.2007 (Universidad Nacional de Córdoba, Argentina) (InfoStat, 2007).

When significant differences were found (p ≤ 0.05), means were compared using Duncan’s multiple range test (α = 0.05). Germination percentages (p) were arcsine-square root transformed before analysis, in accordance with the methods of Zar (1999) and Enríquez (2002) (Equation 3):

(3) Germinatión %=arcsen√p

## 3 Results

### 3.1 Pre-experimental stage

#### 3.1.1 Lethal temperature determination

**Figure 1:**
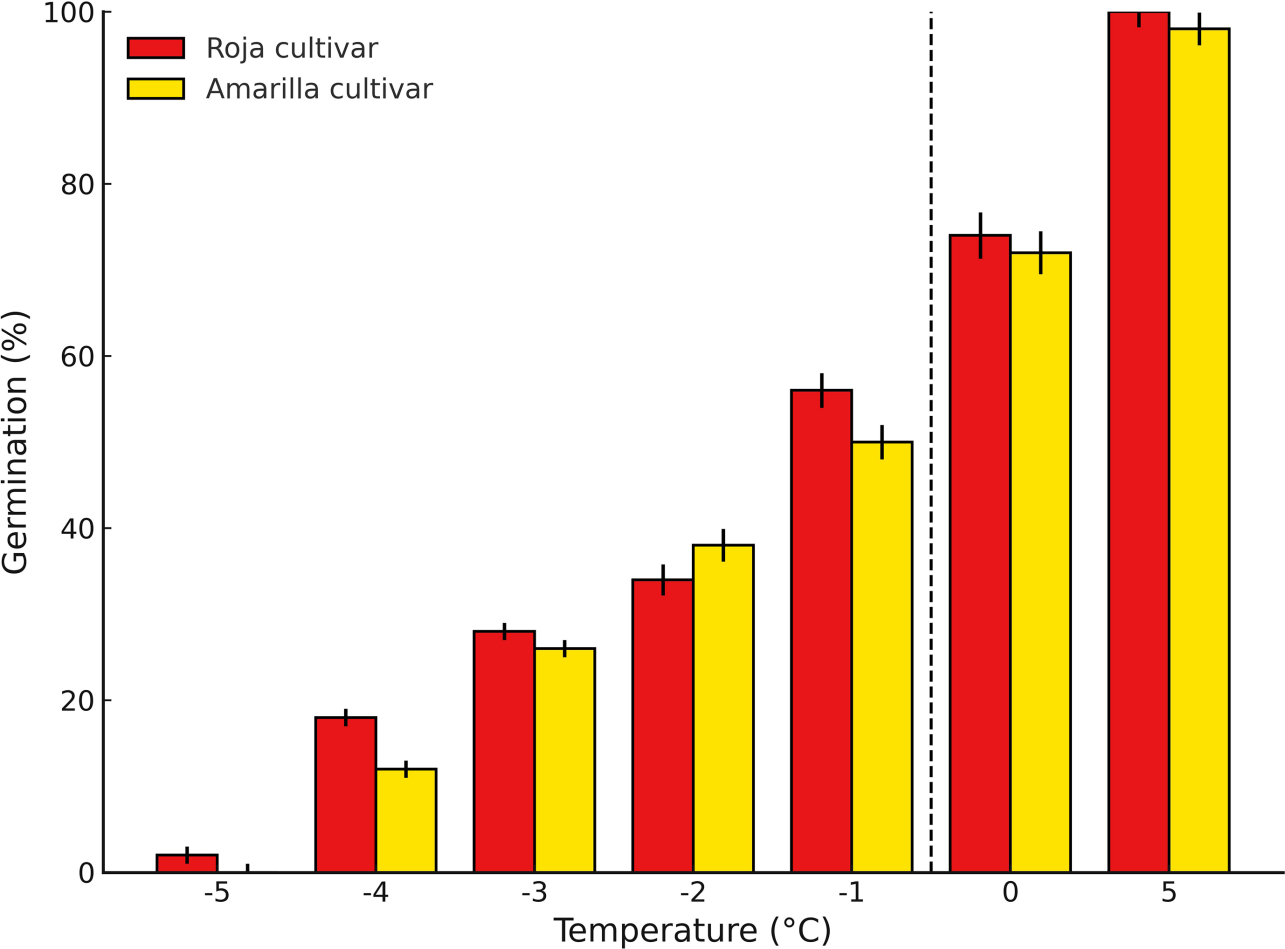
Germination response of Roja cultivar and Amarilla cultivar across a temperature gradient. Bar plots show mean germination values (%) ± standard error (SE) at the tested temperatures (−5, −4, −3, −2, −1, 0 and 5 °C). Roja cultivar is represented in red bars, while Amarilla cultivar is shown in yellow bars. The dashed vertical line highlights the transition before 0 °C, considered a critical threshold for germination.

**Figure 1** shows the impact of low temperatures on seed germination. Exposure to −5 °C completely inhibited Amarilla cultivar germination, while Roja cultivar retained only 3%. Based on these results, −4 °C was defined as the critical threshold for subsequent frost treatments.

#### 3.1.2 Imbibition and absorption kinetics under optimal temperature

**Figure 2:**
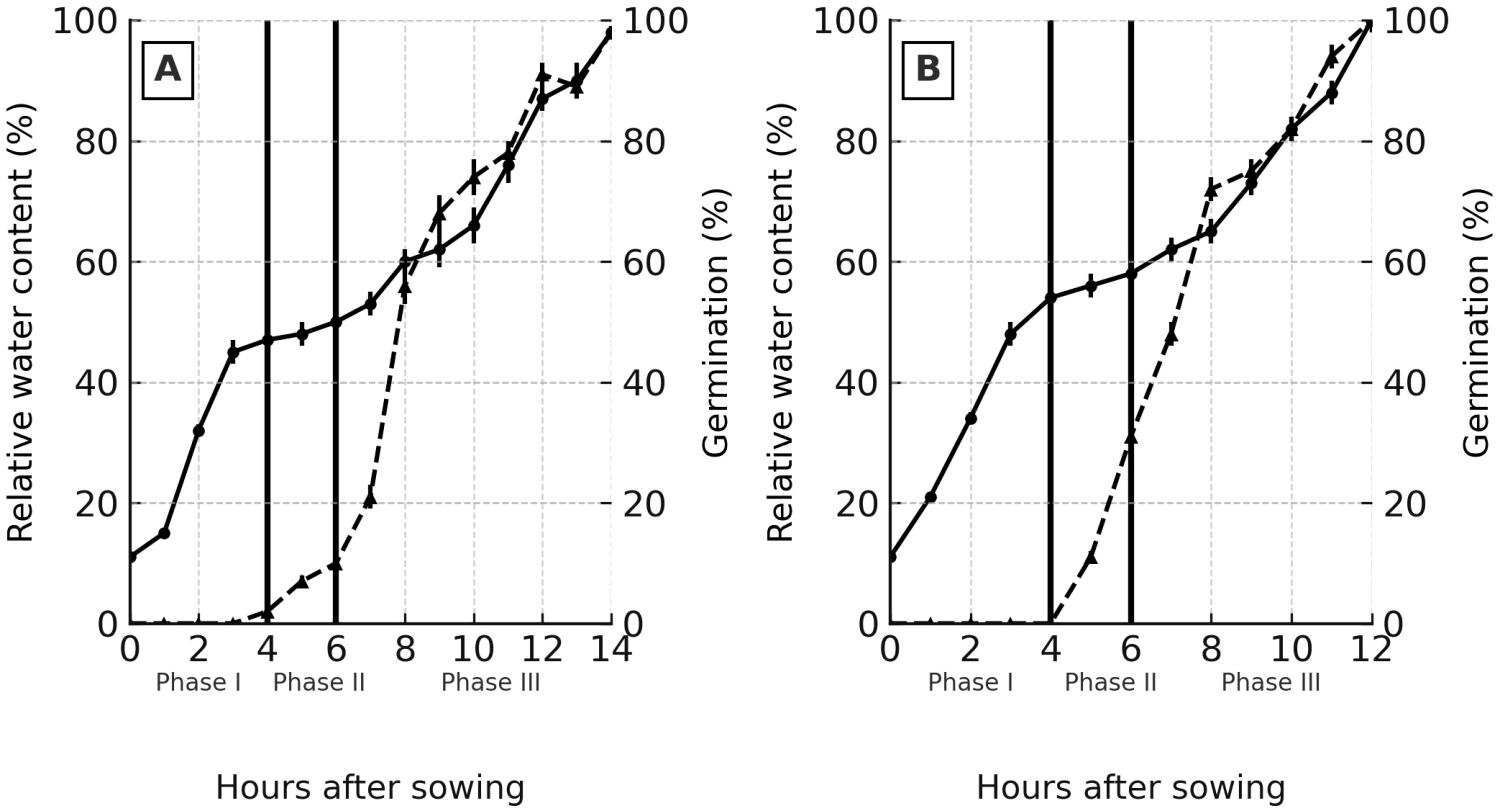
Relative water content and germination in quinoa seeds during early seed water uptake **(A)** Roja cultivar. **(B)** Amarilla cultivar. Relative water content (solid line with circles, left Y-axis) and germination (dashed line with triangles, right Y-axis) are shown as mean values ± standard error (SE). Vertical black lines at 4 h and 6 h indicate the transitions between developmental phases: Phase I (0–4 h), Phase II (5–6 h), and Phase III (>6 h). Germination measurements were obtained at an optimal temperature of 20 °C.

Relative water content (RWC) and germination were monitored to establish the duration of germination phases (**Figure 2**). Both cultivars displayed similar patterns: Phase I lasted 4 h, Phase II lasted 2 h, and Phase III started after 6 h, coinciding with radicle emergence.

### 3.2 Effect of occasional frost on seed water uptake rate

**Figure 3:**
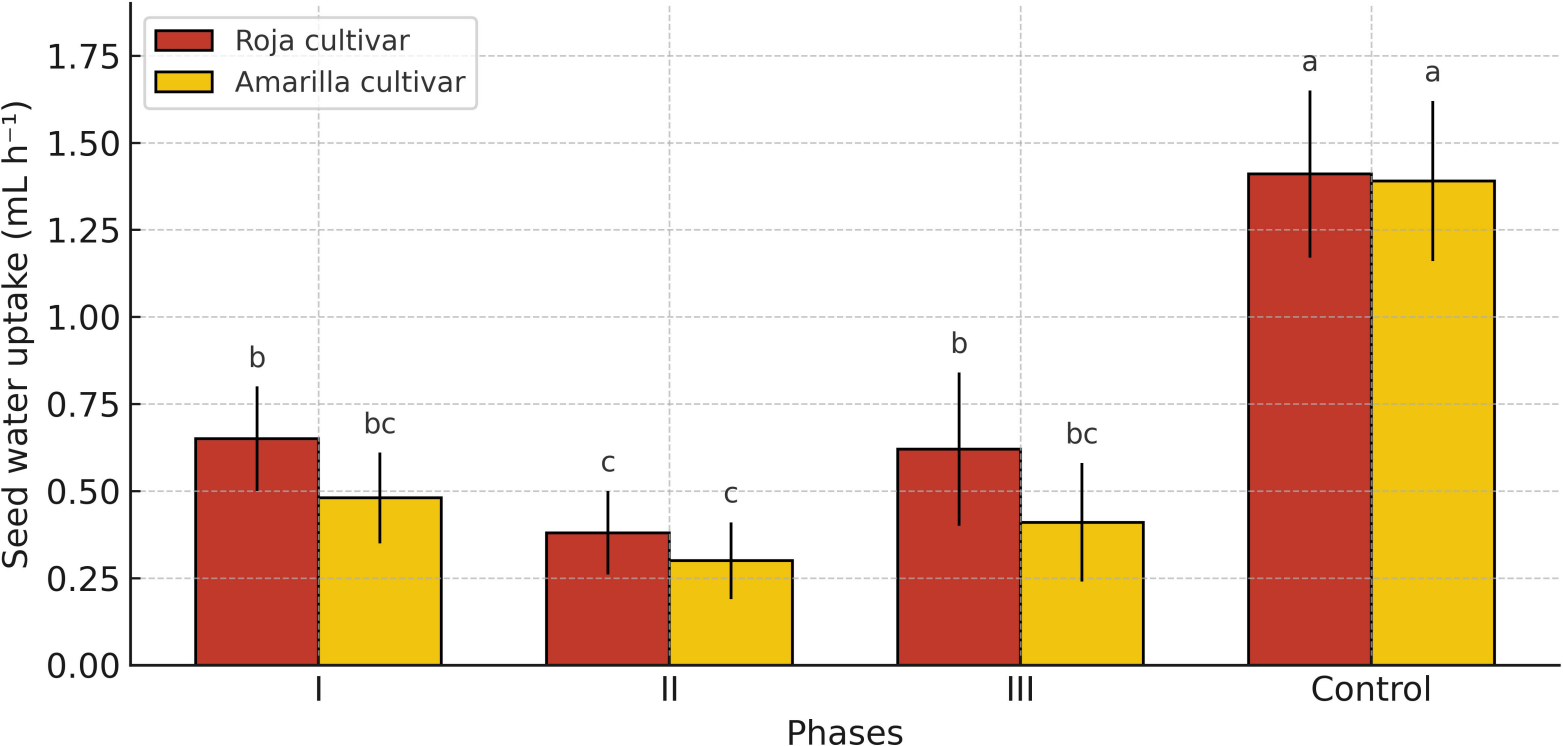
Effect of an occasional frost event (−4 °C to 0 °C) on the seed water uptake rate (Vi) of Roja cultivar and Amarilla cultivar quinoa seeds across different germination phases. Values are means ± SE (n = 5). Different letters indicate significant differences among treatments.

The application of a single frost event (−4 to 0 °C) during seed water uptake significantly reduced Vi compared with the control (p< 0.05). Frosted seeds absorbed water at a rate of 0.5 mL H_2_O h^-1^, whereas the control reached 1.4 mL H_2_O h^-1^ (**Figure 3**).

### 3.3 Effect of occasional frosts on the different stages of germination

**Figure 4:**
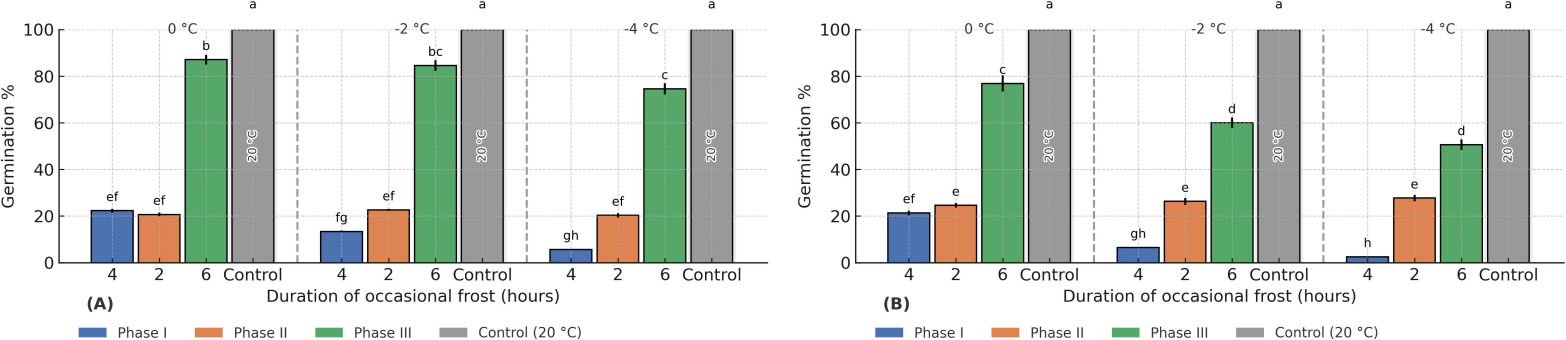
Final germination percentage with occasional frost applied at different germination stages in the Red **(A)** and Yellow **(B)** cultivars. Different letters indicate significant differences between treatments (p ≤ 0.05). Each panel represents a different temperature treatment (0 °C, −2 °C, −4 °C). Colors indicate the germination phase during which frost was applied: Phase I (blue), Phase II (orange), Phase III (green), and the 20 °C control (grey).

In the Roja cultivar, exposure to frost during Phases I and II resulted in a marked decrease in germination, with losses approaching 80% compared with the control across all tested temperatures (0, −2, and −4 °C). The most severe effect occurred when frost at −4 °C was applied in Phase I, producing a 95% reduction in final germination (**Figure 4A**). When frost occurred in Phase III, germination also decreased significantly, although less drastically than in Phases I and II. The magnitude of the reduction increased as temperature decreased, with declines of 15% at 0 °C, 15% at −2 °C, and 25% at −4 °C.

The Amarilla cultivar exhibited a similar pattern, showing strong sensitivity across all germination phases (**Figure 4B**). At −2 and −4 °C, germination was severely impaired particularly during Phases I and II. In Phase I, losses reached 93.5% and 97% at −2 °C and −4 °C, respectively, while Phase II showed reductions of comparable magnitude. During Phase III, germination was less affected, but reductions were still substantial, reaching 40% and 50% at −2 °C and −4 °C, respectively.

These results highlight the extreme vulnerability of the early germination stages, especially the first six hours after seed water uptake begins—when membrane integrity and metabolic reactivation are still incomplete, rendering seeds highly susceptible to freezing damage.

### 3.4 Effect of occasional frosts on germination velocity index

**Figure 5:**
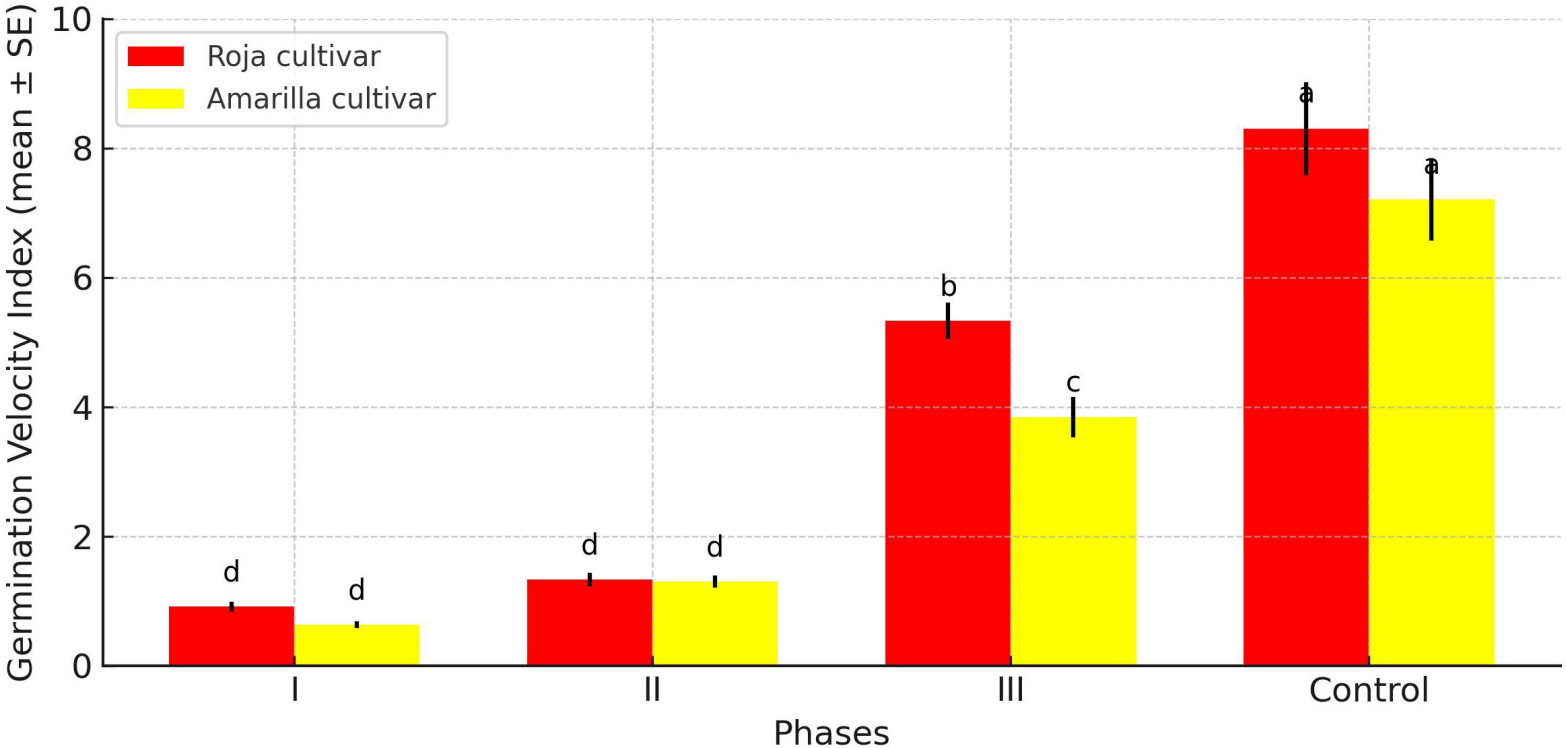
Effect of an occasional frost between 0 and –4 °C on the Germination Velocity Index (GVI) during the germination phases of the Roja and Amarilla quinoa cultivars. Values are means ± SE. Different letters indicate significant differences between treatments (p ≤ 0.05).

At 20 °C, the control treatment had the highest germination rate, completing germination in less than 12 hours (**Figure 5**). In contrast, both cultivars exposed to frost during Phases I and II showed a significant reduction in GVI, with values below 1.3 seeds h^-1^ (p > 0.05 between cultivars). Under these conditions, total germination took more than 38 hours, which was significantly longer than in the control (p ≤ 0.05).

During Phase III, differences between the cultivars became evident. Roja reached a significant higher rate of 5.3 seeds h^-1^ than the Amarilla cultivar, which produced 3.84 seeds h^-1^ (p ≤ 0.05).

Occasional frosts during Phases I and II caused significant reductions in GVI in Roja cultivar (**Table 2**). At 0 °C, germination was delayed by 28 and 26 hours, respectively, in phases I and II, compared with the control. Severe temperatures of –2 and –4 °C during phase I produced extremely low GVI values (<0.5 seeds h^-1^), extending total germination time beyond 96 hours. Phase III showed higher GVI values at all temperatures, suggesting physiological recovery once radicle emergence began. These results demonstrate that the stage of early seed water uptake is the most sensitive to frost.

**Table 2:** Proline content in quinoa seeds subjected to occasional frost during germination.

Timing of application of occasional frosts	Treatment	Roja Proline (µg g^-1^)	Significance (p≤ 0,05)	Amarilla Proline (µg g^-1^)	Significance (p≤ 0,05)
Phase					
I	Control	40,25	f	40,15	f
II		42,11	f	41,23	f
III		41,98	f	40,91	f
I		48,6	cde	45,16	de
II	0 °C	48,1	cde	40,69	f
III		48,41	cde	44,78	e
I		51,48	c	49,88	c
II	-2 °C	63,87	a	49,4	cd
III		57,19	b	50,69	c
I		48,65	cde	52,52	c
II	-4 °C	63,48	a	52,46	c
III		62,64	a	58,37	b

Amarilla cultivar showed a similar pattern (**Table 3**), though with some differences in magnitude. At 0 °C, the strongest reduction occurred in phase II (with a total germination time of 46 h), while phases I and III showed the lowest GVI values (>96 h) at –2 and –4 °C, respectively. During phase III, Amarilla cultivar maintained the highest GVI values, though it was more sensitive to temperature decreases than Roja cultivar (20 hours at –4 °C versus 15 hours at –2 °C).

**Table 3:** Effect of occasional frost on the Germination Velocity Index (GVI) of Roja and Amarilla cultivars.

Cultivar	Time of frost application	Temperature	GVI	%	Germination time	Significance (p≤ 0,05)
	Phase		(seeds/hour)		Hour	
Roja		Control	8,3	16,7	6	a
	I		1,49	2,98	34	e
	II	0 °C	1,54	3,08	32	e
	III		6,75	13,5	7,4	b
	I		0,44	0,88	>96	f
	II	-2 °C	1,3	2,6	38,4	e
	III		5,8	11,6	8,6	c
	I		0,36	0,72	>96	f
	II	-4 °C	1,14	2,28	43,8	e
	III		3,44	6,88	14,5	d
Amarilla		Control	7,19	14,38	7	a
	I		1,29	2,58	39	e
	II	0 °C	1,08	2,16	46	f
	III		5,6	11,2	9	b
	I		0,44	0,88	> 96	g
	II	-2 °C	1,28	2,56	39	e
	III		3,4	6,8	15	c
	I		0,16	0,32	>96	g
	II	-4 °C	1,53	3,06	28	e
	III		2,53	5,06	20	d

The table shows the mean GVI values (seeds·h^-1^), the standard error (SE), and the total number of hours required to complete germination after the treatment. The “%” column indicates the percentage decrease in final germination compared with the control, calculated for each phase–temperature combination. Different letters in the significance column indicate statistical differences among treatments (p ≤ 0.05).

Both cultivars showed a similar pattern: extreme sensitivity in phases I and II, followed by partial recovery in phase III. However, Amarilla cultivar was consistently more affected than Roja cultivar by severe frost. For instance, at –4 °C in phase III, Roja reached 3.44 seeds h^-1^, while Amarilla reached only 2.53 seeds h^-1^. These results suggest that Roja has slightly higher resilience to freezing stress during late germination.

### 3.5 Effect of occasional frosts on osmotic potential

**Figure 6:**
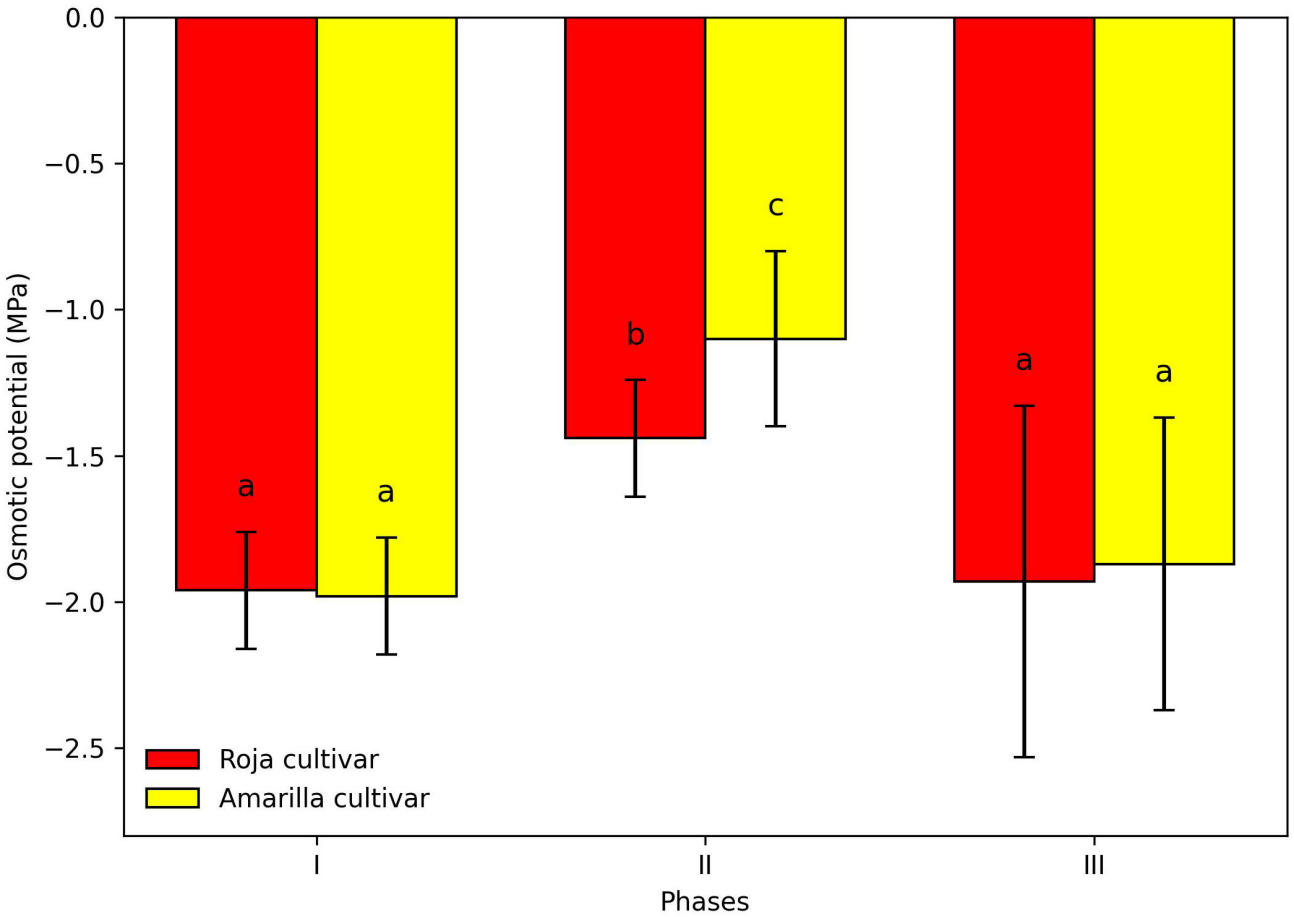
Effect of an occasional frost between –4 and 0 °C on the osmotic potential of quinoa during the germination phases of the Amarilla and Roja cultivars. Values are means ± SE. Different letters indicate significant differences among treatments (p ≤ 0.05).

Occasional frost events between −4 and 0 °C significantly decreased the osmotic potential of seeds (**Figure 6**). The only phase that showed a significant reduction was Phase II, which coincides with the period of maximum water uptake (**Figure 2**). Phases I and III did not differ statistically from each other. Overall, the Amarilla cultivar was more sensitive, exhibiting lower osmotic potential values than the Roja cultivar across all phases.

### 3.6 Effect of occasional frosts on proline accumulation

Additionally, the combined patterns observed in imbibition rate, osmotic potential, and proline accumulation suggest that quinoa seeds respond to frost by making coordinated physiological adjustments. The significant decrease in osmotic potential during Phases II and III, accompanied by enhanced proline accumulation, points to a dynamic osmotic adjustment strategy. A significant decrease in osmotic potential was observed only during Phase II. In Phase III, osmotic potential values were numerically lower than those of Phase I, but without statistical differences between phases, which may suggest a trend toward a maintained osmotic adjustment under frost exposure. This pattern occurs in both cultivars but differs in magnitude depending on temperature and phase.

As temperatures decreased, proline concentration increased progressively (**Table 2**). At 0 °C, no significant differences were observed among the germination phases. At −2 and −4 °C, proline content increased sharply, especially during Phases II and III. For the Roja cultivar, the maximum value was recorded at −2 °C in Phase II (63.87 µg g^-1^). The Amarilla cultivar was detected at −4 °C in Phase III (58.37 µ g^-1^).

Based on the results obtained, it can be established that temperatures below 0 °C increased proline content compared to the control (p ≤ 0.05) (**Table 2**). The highest synthesis was observed at −4 °C when applied during Phases II and III in the Roja cultivar. The maximum content was detected in Phase III in the Amarilla cultivar.

## 4 Discussion

Analyzing the effects of low temperatures (−4, −2, and 0 °C) on germination, this study shows that frost occurring during Phases I and II greatly reduces germination, while Phase III maintains the highest germination percentages. This response is partly dependent on the degree of embryo development. After six hours at 20 °C, the radicle emerges and activates physiological defense mechanisms, such as osmolyte accumulation, increased ATPase activity, elevated gibberellin levels, decreased ABA levels, and starch mobilization (Salisbury and Ross, 1992; Bove et al., 2001). **Table 4** confirms that the lowest germination percentage occurred in Phases I and II at all three temperatures for both cultivars. This indicates that exposure to ≤ 0 °C during the first six hours of imbibition induces mortality in over 72% of quinoa seeds.

**Table 4:** Percentage decrease in seed germination of the Roja and Amarilla cultivars under three temperatures during the germination phases.

Cultivar	Frost application phase	Temperature tested (°C)					
		0° (%)	Significance (p ≤ 0,05)	-2° (%)	Significance (p ≤ 0,05)	-4° (%)	Significance (p ≤ 0,05)
Roja	I	78	de	86,6	ef	94,3	fg
	II	80	de	77,5	de	79,8	de
	III	12,9	a	15,5	ab	25,2	b
Amarilla	I	78,6	de	93,5	fg	97	g
	II	75,2	d	73,8	d	72,3	d
	III	23,2	b	40	c	49,5	c

Different letters indicate differences between treatments (p ≤ 0.05).

Although the seeds viability was not verified using biochemical assays, discrepancies between seed viability and post-stress germination have been linked to the induction of secondary dormancy in other studies. In quinoa, exposure to unfavorable or fluctuating conditions, such as chilling, low oxygen levels, or desiccation, can trigger a reversible dormancy even in non-dormant lots (Ceccato et al., 2011; Romero et al., 2018). In the present experiment, the reduction in germination percentage under frost exposure indicates physiological damage rather than secondary dormancy. This is evidenced by the rapid (<4 hours) and synchronous germination of both seed types under control conditions and the absence of dormancy evidence at the beginning of the assays. Additionally, after the application of the occasional frosts, optimal temperatures were applied, resulting in a germination response and other parameters in response to this environmental stimulus.

Although germination assays were conducted at sea level under strictly controlled laboratory conditions, it is important to consider whether the environment in which the seeds were produced may have influenced their physiological responses, particularly with regards to temperature. The Roja and Amarilla cultivars originated from high-altitude quinoa germplasm in the Andean Altiplano (approximately 3800 m a.s.l.) and were cultivated at 1000 m.a.s.l. in the desert. Under these conditions, frost events, sharp diurnal thermal fluctuations, and high solar radiation exert strong selective pressure (Bazile et al., 2016; Hinojosa et al., 2018).

**Table 4** shows that the highest freezing sensitivity occurs during the early imbibition phases (Phases I and II) for both cultivars, where the reductions in germination are statistically significant across all sub-zero temperatures.

In Phase III, Amarilla exhibits a numerically greater reduction than Roja; however, these differences are not statistically significant and should therefore be interpreted as a trend rather than as evidence of lower freezing tolerance at this later stage.

These results indicate that freezing sensitivity during the initial imbibition phases can strongly affect crop establishment under frost events, while cultivar differences become more noticeable only in later stages of germination.

The Amarilla and Roja cultivars used in this study originate from highland quinoa populations (~3800 m a.s.l.) that were later propagated for several generations under desert lowland conditions (~1000 m a.s.l.). This combined highland–lowland background may contribute to their divergent physiological responses to freezing, supporting the hypothesis that cold tolerance in quinoa reflects both genetic origin and local adaptation.

These patterns are consistent with previous studies showing that the initial hydration phases are the most susceptible to freezing stress. Exposure to temperatures near or below 0 °C during initial water uptake disrupts membrane integrity and metabolic activation, resulting in irreversible cellular injury (Bewley et al., 2013). Similar physiological vulnerability during early imbibition has been documented in several species and is considered a universal mechanism associated with incomplete membrane repair and high metabolic sensitivity.

Studies in legumes demonstrate that low temperatures impair membrane reorganization, enzymatic activity, and the expression of stress-response genes, delaying radicle protrusion (Bhat et al., 2022). Comparable reductions in germination speed due to membrane damage and metabolic slowdown have been reported in soybean under chilling stress (Szczerba et al., 2021). In quinoa, temperatures close to 0 °C markedly slow germination, and nocturnal frosts as low as –6 °C substantially delay emergence (Bois et al., 2006). These findings support our results: early water uptake is the most vulnerable stage, and the ability to recover increases once the radicle emerges.

Sensitivity during early hydration in quinoa has also been reported under chilling and freezing conditions, with high mortality observed when seeds are exposed to sub-zero temperatures before radicle protrusion (Hinojosa et al., 2018). Evaluations of frost response in quinoa seedlings show that freezing injury results from intracellular ice formation and impaired osmotic adjustment, particularly in cultivars not adapted to high-altitude environments (Jacobsen et al., 2007).

Genotypic differences in cold tolerance among quinoa cultivars are widely documented. In the present study, both cultivars share a highland origin; therefore, the greater numerical sensitivity observed in Amarilla at later germination stages reflects cultivar-specific variation. Such differences are well recognized in quinoa breeding and ecophysiology, where tolerance to low temperatures is strongly associated with each cultivar’s genetic background and local agroecological history (Zurita-Silva et al., 2014; Bazile et al., 2016; Murphy and Matanguihan, 2015). Taken together, these findings suggest that frost events during the initial hours of seed hydration can severely hinder stand establishment, and that cultivar-specific responses should be considered when selecting quinoa for environments prone to freezing.

Differences between the ancestral high-altitude environment and the intermediate-altitude production conditions may contribute to variation in seed physiology. Maternal environmental effects modify key seed traits, such as dormancy level, germination rate, and stress tolerance, through their influence on temperature, photoperiod, and altitude-related climatic variation during seed development. Penfield and MacGregor (2017) demonstrated that these factors can significantly alter dormancy depth and subsequent germination performance. Additionally, Klupczyńska and Pawłowski (2021) reported that the maternal environment affects the regulation of hormones and genes associated with stress responses.

Therefore, the germination behavior observed in the Roja and Amarilla cultivars likely reflects an interaction between their high-altitude genetic background and their more recent propagation under intermediate-altitude desert conditions. This context is particularly relevant when interpreting their responses to simulated frost because the combined influence of genotype, maternal environment, and production altitude may affect early seed performance and partially explain the patterns observed in this study.

However, the possibility that transient exposure to subzero temperatures could modulate the hormonal balance between abscisic acid and gibberellins, thereby inducing partial secondary dormancy, cannot be discounted at this time. Subsequent research endeavors, such hormonal profiling, are expected to provide further insight into this aspect.

Cold stress induces water and osmotic stress in seeds, which promotes the accumulation of compatible solutes, such as proline (Szabados and Savouré, 2010). These metabolites play a central role in protecting cells by stabilizing membranes and proteins, reducing osmotic potential, and facilitating water retention (Hayat et al., 2012; Renzetti et al., 2024). In our study, proline content increased under subzero temperatures, particularly during Phases II and III. In Roja, the increase in proline is more pronounced under severe frost. The maximum proline accumulation occurred at −2 °C in Phase II (63.87 μg g^-1^), suggesting a robust osmotic adjustment response at this stage. In Amarilla, the highest value occurred at −4 °C in Phase III (58.37 μg g^-1^). Although Amarilla also showed increased proline under frost stress, the magnitude was generally lower than that of Roja during the most critical phase (Phase II). This suggests a cultivar−specific difference in osmotic adjustment mechanisms. This finding is consistent with recent reports identifying proline as a key metabolic marker of cold tolerance in quinoa and permit use this like a biomarker (Zheng et al., 2022; Fu et al., 2025; Dehghanian et al., 2024).

Zhu et al. (1997) demonstrated that temperatures below 0 °C result in cellular dehydration and a decrease in osmotic potential below −1.1 MPa. These conditions can literally “dry out” the cell if not corrected. Thus, the higher proline content observed in our quinoa seeds confirms the occurrence of water–osmotic stress induced by frost and supports its role in osmotic adjustment.

**Table 5** shows relationship between imbibition rate, osmotic potential, and proline content. The general imbibition pattern was not altered in the presence of frost, but the total amount of water absorbed by the seeds decreased. This was reflected by a reduced imbibition rate (Vi), particularly in Phases I and III, though the classical three-phase sequence remained intact (Salisbury and Ross, 1992). Meanwhile, the osmotic potential (Φ) became more negative in the phases with higher water uptake. This is consistent with the accumulation of osmolytes such as sugars (e.g., sucrose and fructose) and amino acids (e.g., proline and betaine), which lower the osmotic potential without interfering with cellular metabolism (Aslam et al., 2022).

**Table 5:** Effect of occasional frost on germination, Vi, Φ, proline content and RWC in quinoa seeds.

Cultivar	Time of frost application	Water absorption uptake speed	Significance (p≤ 0.05)	Osmotic potential	Significance (p≤ 0.05)	Proline	Significance (p≤ 0.05)	Relative water content	Significance (p≤ 0.05)
	Phase	(mL/hours)		(MPa)		(µg g-1)		(mL/seeds)	
Roja	I	0,73	a	-1,96	c	49,58	a	0,078	a
	II	0,38	c	-1,51	b	48,10	ab	0,015	c
	III	0,62	b	-1,93	c	56,08	a	0,074	a
Amarilla	I	0,48	bc	-1,98	c	49,19	ab	0,039	b
	II	0,30	c	-1,01	a	40,69	b	0,012	c
	III	0,41	c	-1,87	c	51,28	a	0,049	b

Different letters indicate differences between treatments (p ≤ 0.05).

In Phase II, despite lower water content, osmotic potential was less negative, indicating a better balance between water influx and solute accumulation. This adjustment prevents dehydration and prepares the embryo for radicle emergence in Phase III. According to Bradford (1995), the amount of water absorbed in Phase II is sufficient for germination to be completed, so stress applied after this stage has less impact on seed viability.

### 4.1 General implications

Furthermore, our findings are consistent with prior research on quinoa frost resistance (Squeo et al., 1991; Jacobsen et al., 2007) by confirming that tolerance is not only stage-dependent but also cultivar-dependent. The contrasting behavior of Amarilla and Roja cultivars highlights the importance of genetic diversity within quinoa germplasm. From a physiological perspective, the interplay between osmotic potential regulation and proline accumulation appears central to maintaining cellular integrity under freezing stress. These findings also align with the broader literature on stress physiology, which suggests that compatible solutes act as metabolic buffers during abrupt temperature fluctuations (Hayat et al., 2012; Renzetti et al., 2024). Finally, the Amarilla cultivar’s higher germination in Phase III highlights its potential as a donor genotype in breeding initiatives targeting environments with recurrent early frosts.

Recent studies confirm that cold tolerance in quinoa involves an integrated response of osmotic adjustment, redox regulation, and the activation of antioxidant defenses (Zheng et al., 2022; Fu et al., 2025). Proline accumulation, along with soluble sugars, is recognized as a key adaptive trait in response to frost. It contributes to both water retention and the mitigation of oxidative stress (Jacobsen et al., 2003; Renzetti et al., 2024).

From an applied perspective, our findings underscore the critical sensitivity window during the first six hours of imbibition. Moreover, the greater tolerance of the Amarilla cultivar in Phase III indicates the presence of genetic variability that can be utilized in breeding programs. Management strategies such as osmopriming or cold priming could improve quinoa seedling establishment in areas prone to early-season frosts.

## 5 Conclusions

In summary, this work contributes new evidence that occasional frosts impose stage-specific constraints on quinoa germination and that cultivar significantly impacts establishment success. The integrative analysis of water uptake, osmotic potential, and osmolyte accumulation highlights the importance of physiological markers, such as proline, for assessing tolerance. These insights could make practical recommendations, such as adjusting sowing dates or using priming techniques, as well as long-term strategies in breeding programs to promote resilience. Future research should further elucidate the molecular basis of these responses and extend the evaluation to include additional Chilean and international germplasm to ensure that quinoa can continue to thrive in the increasingly variable climate scenarios of the Andes and beyond.

The present study demonstrates that both Amarilla and Roja quinoa cultivars can tolerate occasional frost events during germination. However, their germinative capacity is significantly reduced when frost occurs during the first two phases (from sowing up to 6 hours after imbibition). In contrast, applying frost during Phase III resulted in higher germination rates, exceeding 75% for Amarilla and 50% for Roja.

No lethal effects of temperatures at 0, −2, and −4 °C were observed on germination capacity in either cultivar. However, seeds exposed to frost during Phases I and II showed the greatest sensitivity, with Phase I being the most vulnerable. In Phase III, both cultivars maintained a germination rate above 50%. Amarilla consistently displayed greater tolerance to occasional frost than Roja.

## Data Availability

The raw data supporting the conclusions of this article will be made available by the authors, without undue reservation.
