# Supplementary material for: Tolerance to occasional frosts during germination in Chilean Altiplano quinoa (Chenopodium quinoa Willd.) cultivars
Source: Front Plant Sci. 2026 Jan 26;16:1718308. doi: 10.3389/fpls.2025.1718308 (PMC12884543; doi:10.3389/fpls.2025.1718308)
Supplement: Supplementary file 1 [file SupplementaryFile1.pdf]

Determination of lethal temperature and exposure time for each germination phase

The quinoa seeds were placed in Petri dishes containing a 1:1:1 mixture of peat, sand, and perlite. Then, 25 g of the mixture was added to each dish, and the dishes were then moistened with 20 ml of distilled water. This process was carried out using a 20 ml volumetric pipette. To prevent humidity exchange between the Petri dishes and the cold chamber atmosphere, the dishes were covered. The relative humidity within the chamber ranged from 45 % to 49 %, while that within the dishes ranged from 53% to 58%. These conditions simulated a “black frost,” whereby the substrate temperature remains above the air dew point, avoiding condensation and allowing ice to form exclusively on the substrate surface.

The effect of low temperatures was evaluated for each of the three germination phases (4, 2, and 6 hours, respectively) under optimal conditions (20 °C). For each phase, 50 seeds of the Roja and Amarilla cultivars were exposed to frost at 0 °C, −2 °C, and −4 °C for the corresponding duration, after which they were allowed to recover at 20 °C until 100 % germination was reached under control conditions.

Specifically, during Phase I (imbibition), the seeds were exposed directly to temperatures of 0 °C, −2 °C, and −4 °C for four hours, after which they were transferred to 20 °C. In Phase II (the lag phase), the seeds were first germinated at 20 °C for the same duration as Phase I. Then, they were exposed to 0, −2, or −4 °C for two hours. Afterwards, they returned to 20 °C. In Phase III (radicle protrusion), the seeds underwent pre-germination for six hours at 20 °C (Phases I and II combined). Thereafter, the seeds were exposed to temperatures of 0, −2, or −4 °C for an additional six hours. Finally, the seeds were returned to an optimal temperature for the remainder of the experiment.

References

Bois, J. F., Winkel, T., Lhomme, J. P., Raffaillac, J. P., & Rocheteau, A. (2006). Response of some Andean cultivars of quinoa (Chenopodium quinoa Willd.) to temperature: effects on germination, phenology, growth and freezing. Eur. J. Agron. 25, 299–308. https://doi.org/10.1016/j.eja.2006.06.007

Jacobsen, S.-E., Monteros, C., Corcuera, L. J., Bravo, L. A., Christiansen, J. L., & Mujica, A. (2007). Frost resistance mechanisms in quinoa (Chenopodium quinoa Willd.): short communication. Eur. J. Agron. 26, 471–475. https://doi.org/10.1016/j.eja.2007.01.006

Bradford, K. J. (1995). Water relations in seed germination. In Kigel, J. & Galili, G. (Eds.), Seed Development and Germination, pp. 351–396. Routledge. https://doi.org/10.1201/9780203740071

Squeo, F. A., Rada, F., Azócar, A., & Goldstein, G. (1991). Freezing tolerance and avoidance in high tropical Andean plants. Oecologia 86, 378–382. https://doi.org/10.1007/BF00317604

Sierra-Almeida, A., Cavieres, L. A., & Bravo, L. A. (2009). Freezing resistance varies within the growing season and with elevation in high-Andean species of central Chile. New Phytol. 182, 461–469. https://doi.org/10.1111/j.1469-8137.2008.02756.x
